# An SPH Approach for Non-Spherical Particles Immersed in Newtonian Fluids

**DOI:** 10.3390/ma13102324

**Published:** 2020-05-19

**Authors:** Nadine Kijanski, David Krach, Holger Steeb

**Affiliations:** 1 Institute of Applied Mechanics (CE), University of Stuttgart, Pfaffenwaldring 7, 70569 Stuttgart, Germany; david.krach@mechbau.uni-stuttgart.de (D.K.); holger.steeb@mechbau.uni-stuttgart.de (H.S.); 2 Stuttgart Center for Simulation Technology, Pfaffenwaldring 5a, 70569 Stuttgart, Germany

**Keywords:** DNS, SPH, solid body motion, contact models, contact forces, repulsive force

## Abstract

Solid particles immersed in a fluid can be found in many engineering, environmental or medical fields. Applications are suspensions, sedimentation processes or procedural processes in the production of medication, food or construction materials. While homogenized behavior of these applications is well understood, contributions in the field of pore-scale fully resolved numerical simulations with non-spherical particles are rare. Using Smoothed Particle Hydrodynamics (SPH) as a simulation framework, we therefore present a modeling approach for Direct Numerical Simulations (DNS) of single-phase fluid containing non-spherically formed solid aggregates. Notable and discussed model specifications are the surface-coupled fluid–solid interaction forces as well as the contact forces between solid aggregates. The focus of this contribution is the numerical modeling approach and its implementation in SPH. Since SPH presents a fully resolved approach, the construction of arbitrary shaped particles is conveniently realizable. After validating our model for single non-spherical particles, we therefore investigate the motion of solid bodies in a Newtonian fluid and their interaction with the surrounding fluid and with other solid bodies by analyzing velocity fields of shear flow with respect to hydromechanical and contact forces. Results show a dependency of the motion and interaction of solid particles on their form and orientation. While spherical particles move to the centerline region, ellipsoidal particles move and rotate due to vortex formation in the fluid flow in between.

## 1. Introduction

Solid bodies immersed in Newtonian fluids can be found in many fields of mechanical, civil and environmental engineering. Coarse-graining and therefore simplifying the multi-phase response of the mixture, suspensions are often constitutively described on a continuum-scale as non-Newtonian fluids with either shear-thinning or shear-thickening behavior [1,2]. Such continuum models present a good approximation when it comes to general investigations of fluid flow of suspensions on length scales significantly larger than the characteristic particle diameter. However there is no information about the microscopical influence of solid–solid contacts as well as hydro-mechanical fluid–solid interactions which could lead to well-known phenomena like de-mixing [3], particle formation like hydroclusters [4,5], shear-wall migration [6,7] and shear-induced particle migration [8]. Theoretical approaches in the field of motion of solid particles immersed in a fluid are presented for example by Jeffery [9,10,11] and Guazzelli and Morris [12], who consider pore-scale effects (microhydrodynamics) as well as continuum approaches. However, to overcome limitations of classical macroscopic continuum formulations, we present a multi-scale modeling approach for fully resolved solid particles immersed in a fluid. The model takes into account a Newtonian carrier fluid coupled with a repulsive force model for the solid–solid contact (calculated from a potential).

Various numerical methods to investigate suspended particles flow in Computational Fluid Dynamics (CFD) exist. Often either a fully or coupled Discrete Element Method (DEM) is used. Thus contributions exhibit Lattice-Bolzmann-DEM approaches [13], Smoothed Particle Hydrodynamic (SPH)-DEM approaches [14,15], Finite-Element-DEM approaches [16,17,18] or Particle-Finite-Element-Method approaches (PFEM) [19] to name only a few. All of these methods have in common, that they are either Eulerian grid-based methods (FEM, LB) which are limited when large deformations are involved or free surface flow is dominant or else are not able to discretize the surrounding fluid without a coupling algorithm (DEM). Thus, streamlines in the vicinity of spherical and especially non-spherical particles can not be analyzed. To overcome these limitations, we present a quasi-incompressible Smoothed Particle Hydrodynamics (SPH) approach. SPH as a meshless Lagrangian method presenting remarkable advantages when it comes to fully resolved solid–fluid interfaces and interactions [5,20]. Thus SPH is the ideal choice to model suspended solid bodies since the carrier fluid as well as the immersed solid particles can be discretized with SPH particles. Therefore, SPH is leading to the possibility of investigating the physical properties of the single particle-fluid interaction and the solid–solid contacts individually. As an example, streamlines in the vicinity of a solid particle can be determined. In addition, the fully resolved modeling approach allows to build rigid solid aggregates of arbitrary forms using solid SPH particles and to discretize the fluid phase with fluid SPH particles. Therefore, in contrast to continuum mixture-theory approaches, each material point P(x,t) is either occupied by the fluid or the solid phase. This renders usage of surface-coupled hydromechanical forces easy and allows to simulate aggregates with complex non-spherical forms. For the implementation of the presented model we use the highly scalable software library HOOMD-blue [21,22] which we extended for SPH and recently validated in terms of scalability on CPU and GPU clusters [23]. A quite similar method for the simulation of suspensions using SPH was presented by Vázquez-Quesada et al. [5,24,25]. They show simulations of dense suspensions and present an analysis of the rheological behavior in terms of shear-thickening and -thinning. However, a detailed analysis of micro-scale effects, related to contact between the solid particles is missing. Further, their investigations are restricted to spherical particles.

Considering solid particle motion in a fluid, suspensions are one possible application. In this case, the Péclet number which represents the ratio of mechanical to Brownian forces characterizes non-colloidal suspensions [26,27]. Simulating only large Péclet numbers, we assume that hydromechanical forces dominate the flow process and Brownian forces, and further colloidal phenomena, can be neglected. Moreover, the non-colloidal character can be defined by larger particle diameters (d≥1μm) and a low aspect ratio between particle volume and particle surface. Therefore, we focus on diameters larger than 1μm and aspect ratios between 0.5 and 0.95.

Similar to Tanner [26,28] and Vázquez-Quesada et al. [5,24,25], most publications consider high concentrated, i.e., dense suspensions using the ideal case of monodisperse spherical particles and neglecting the effect and impact of contact between single solid particles. As discussed by Jeffery [9] and Mueller [29], shape and size of solid particles can have significant influences on the flow behavior of suspensions. Within the present approach, we therefore investigate the close environment of non-spherical solid particles immersed in a carrier fluid. With fully resolved simulations we determine the impact of hydromechanical and contact forces on the motion of particles and the interaction with the fluid phase in the vicinity of particles.

## 2. Theory of Solid Particle Motion in Single-Phase Newtonian Fluid

In general, a suspension can be discretized as a Newtonian or a non-Newtonian carrier fluid with immersed solid particles influencing the flow behavior [1]. The particle (number) volume fraction $\phi^{s}$ is a macroscopical quantity which takes into account the amount of solid particles related to the total volume. It can be computed using the number of entities (e.g., SPH particles) of a constituent $n^{s}$ divided by the total number of SPH particles *N* or, in case of a homogeneous particle discretization, by using the volume $dV^{s}$ occupied by the solid particles divided by a finite control volume dV

(1) $$\begin{array}{c} \phi^{s}=\frac{n^{s}}{N}=\frac{dV^{s}}{dV}\;. \end{array}$$

Considering small (suspension-like) systems with only a few number of solid particles and therefore with a particle volume fraction $\phi^{s}<0.05$, hydromechanical forces as well as contact forces can be observed at each solid particle individually. We therefore derive underlying balance equations, i.e., conservation of mass and momentum, with a constitutive equation for the Cauchy stress tensor.

### 2.1. Balance Equations for Single-Phase Fluid Flow

Suspensions consist of a carrier fluid with immersed solid particles. Using fully resolved numerical simulations, hydromechanical forces are evaluated for both, the solid and the fluid phase, individually. Taking into account solid–solid contact forces as well, a coupled formulation of the momentum conservation including an extra part for solid–solid contact is used. Underlying conservation equations are the balance of mass and the balance of momentum, i.e., the Navier–Stokes equations for quasi-incompressible single-phase bulk fluid flow in local form

(2) $${\dot{\rho }}^{f}+\rho^{f}\;\mathrm{div}\;v_{f}=0\;,$$

(3) $$\rho^{f}\;{\dot{v}}_{f}=\mathrm{div}\;T+\rho^{f}\;g\;,$$

where $\rho^{f}$ is the fluid density, $v_{f}$ is the flow velocity and $\rho^{f}\;g$ are the body forces. T denotes the Cauchy stress tensor which is split into an extra or non-equilibrium part and into a pressure or equilibrium part $T=T_{eq}+T_{neq}$. While the equilibrium part is given by

(4) $$T_{eq}=-p\;I,$$

the non-equilibrium part of the stress tensor is referred to as viscous extra stress

(5) $$T_{neq}=\mu^{f}\;\left(\mathrm{grad}\;v_{f}+{\mathrm{grad}}^{T}\;v_{f}\right)\;,$$

assuming a divergence-free velocity field $v_{f}$. This leads to the updated form of the balance of linear momentum for a homogeneous quasi-incompressible fluid with constant dynamic viscosity $\mu^{f}$

(6) $$\rho^{f}\;{\dot{v}}_{f}=\mu^{f}\;\mathrm{div}\left(\mathrm{grad}\;v_{f}\right)-\mathrm{grad}\;p+\rho^{f}\;g\;.$$

Since the fluid phase is considered to be barotropic, the fluid pressure *p* can be computed as a function of the fluid density $\rho^{f}$ using an equation of state in form of Tait’s equation

(7) $$p=\frac{\rho_{0}^{f}c^{2}}{\gamma }\left[{\left(\frac{\rho^{f}}{\rho_{0}^{f}}\right)}^{\gamma }-1\right]+p_{0},$$

where $\rho_{0}^{f}$ is the initial fluid density, *c* the (numerical) speed of sound and $p_{0}$ is a constant background pressure to avoid negative pressures in the system. Moreover γ=7 is a common choice for quasi-incompressible flow [30,31].

### 2.2. Introduction to Motion of Solid Particles Immersed in a Fluid

When considering solid particles immersed in the fluid phase, underlying conservation equations (Equations (2) and (3)) are also valid for the fluid–solid interaction. Since we consider a surface-coupled approach, no mass exchange between bulk phases at solid–fluid interfaces exist (Figure 2). However, solid–solid interactions, i.e., contact forces between two solid particles are described by a repulsive force approach [32]

(8) $$F_{ab}^{\mathrm{rep}}=F_{0}\;\frac{\tau \;e^{-\tau s}}{1-e^{-\tau s}}\;x_{ab}.$$

This commonly used constitutive equation for solid–solid interactions computes a force acting alongside the vector $x_{ab}$ that connects the center of masses of solid bodies $P^{a}$ and $P^{b}$. $F_{0}$ denotes the magnitude of the repulsive force (dependent on the considered boundary value problem), $\tau^{-1}$ sets the active range of the force and *s* is the distance between the surfaces of solid bodies $P^{a}$ and $P^{b}$. The repulsive force $F_{ab}^{\mathrm{rep}}$ is independent of the surrounding fluid and considered by an additional term in the balance equation of linear momentum (Equation (6)) which therefore can be expressed by means of a whole-domain formulation

(9) $$\rho^{\alpha }{\dot{v}}_{\alpha }=\mu^{\alpha }\;\mathrm{div}\left(\mathrm{grad}\;v_{\alpha }\right)-\mathrm{grad}\;p+\rho^{\alpha }g+F^{\mathrm{rep}}$$

with α={s,f} for the solid and the fluid phase, respectively.

## 3. Numerical Modeling of Fluid Flow with Suspended Particles Using SPH

For the purpose of studying the effects of heterogeneous particles in a single-phase fluid, we employ Direct Numerical Simulations (DNS). Our method of choice is Smoothed Particle Hydrodynamics (SPH) [33,34] using an implementation that has previously been shown to accurately reproduce effective hydraulic properties of porous media [35,36,37]. In particular, our implementation incorporates the SPH scheme proposed in [20] together with the boundary conditions proposed in [38]. It is implemented using the highly optimized Molecular Dynamics tool HOOMD-blue [21,22]. The implementation targets both CPU and GPU (cluster) computation and was verified for several representative test cases performed on the supercomputers Hazel Hen (HLRS, Germany) and BinAC (Tübingen, Germany) [23]. The use of SPH is motivated by the fact that we consider suspended solid bodies of arbitrary form instead of spherical solid particles. The meshless nature of SPH renders the discretization of this heterogeneous solid particles comparatively trivial. Moreover, the meshless particle character gives the opportunity to handle interfaces between different phases, i.e., solid and fluid bulk phases, and evaluate forces (contact forces) directly where they appear. Given the Lagrangian nature of SPH, implying that nonlinear convective terms are not required to be modeled, SPH is rather stable at finite Reynolds numbers. Therefore simulations up to Re = 1000 are feasible. Since a detailed study of the numerical scheme is beyond the scope of this contribution, the reader is referred to [33,39] for technical details concerning details of the implementation.

The numerical implementation and therefore discretization of introduced equations results in four cases of SPH particle interaction, which will be dealt with further below: A. Fluid particle with fluid particle, B. Fluid particle with a non-moving solid particle, C. fluid particle with a moving solid particle and D. Moving solid particle with moving solid particle.

### 3.1. SPH for Single-Phase Fluid (Case A and B)

In SPH [40], the discretization of governing partial differential equations gives rise to a set of interacting collocation points (referred to as SPH particles). At each unstructured integration point, every field function f(x) can be represented by local interpolation using convolution with the Dirac delta function

(10) $$\begin{array}{cc} f(x)\; & =\;\int_{\Omega }f\left(x^{\prime }\right)\;\delta \left(x-x^{\prime }\right)\;dv. \end{array}$$

**Figure 1:**
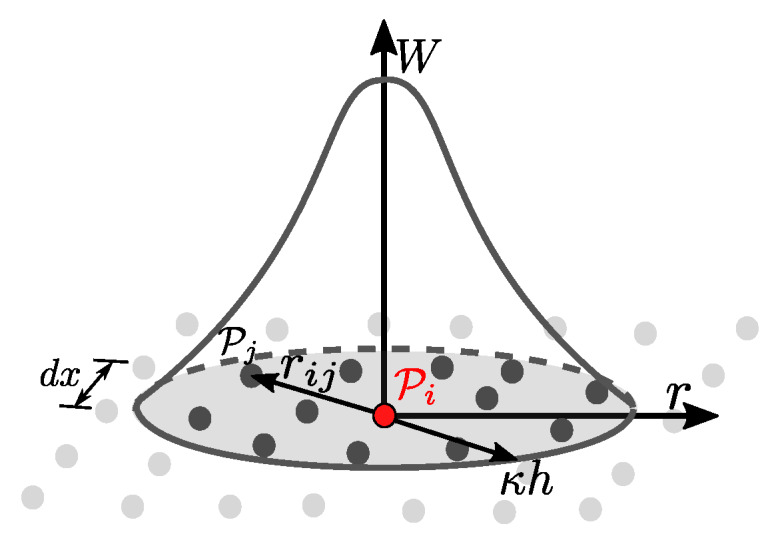
Representation of the kernel function *W* with compact support κh and dependent on particle distance $r_{ij}$.

The Dirac delta function $\delta (x-x^{\prime })$ is replaced by a kernel function *W* with compact-support and smoothing length *h* (Figure 1) leading to an approximation of the field function f(x)

(11) $$\begin{array}{cc} f(x)\; & \approx \;\int_{\Omega }f\left(x^{\prime }\right)\;W\;\left(x-x^{\prime },h\right)\;dv. \end{array}$$

Since SPH is a particle method, the kernel representation is converted into a spatial discretization and therefore every field function transforms into a particle property $f_{i}=f\left(x_{i}\right)$. At point $P_{i}$ the property $f_{i}$ is computed by summation over function values of all neighboring particles $P_{j}$ with particle volume $V_{j}$

(12) $$\begin{array}{cc} f_{i}\; & =\;\sum_{j}^{N}f_{j}\;W\;\left(x_{i}-x_{j},\;h\right)\;V_{j}\;. \end{array}$$

Moreover continuous differential operators can be discretized in the same way and hence transform into viscous and pressure short-range interaction forces $F_{ij}^{V}$ and $F_{ij}^{P}$, respectively.

Following this, the local force balance resulting from the discretization of the local balance of momentum (Navier–Stokes Equation (6)) can be expressed as

(13) $$m_{i}{\dot{v}}_{i}=F_{i}^{SPH}=\sum_{j}F_{ij}^{V}-\sum_{j}F_{ij}^{P}+F_{i}^{B}.$$

The shown discretization applies to discrete integration points $x_{i}$, referred to as SPH particles $P_{i}$, of mass $m_{i}$ and subject to the advection particle velocity $v_{i}$. All forces acting on SPH particle $P_{i}$ can be considered as a combined force $F_{i}^{\mathrm{SPH}}$. This contains the volumetric force on a particle $F_{i}^{B}=m_{i}g$ as well as the pairwise dissipative viscous interaction forces $F_{ij}^{V}$ and the pairwise conservative pressure interaction forces $F_{ij}^{P}$ both acting between particle $P_{i}$ and its nearest neighbor particles $P_{j}$. They are discretized by [41]

(14) $$F_{ij}^{V}=\left\{\begin{array}{c} \left[\frac{1}{n_{i}^{2}}+\frac{1}{n_{j}^{2}}\right]\frac{2\mu_{i}\mu_{j}}{\mu_{i}+\mu_{j}}\frac{v_{i}-v_{j}}{r_{ij}}\frac{\partial W_{ij}}{\partial r_{ij}}\;\mathrm{if}\;x_{j}\in \Omega_{f}\;\mathrm{and}\;\forall x_{i}\in \Omega_{f},\\ \left[\frac{1}{n_{i}^{2}}+\frac{1}{n_{j}^{2}}\right]\frac{2\mu_{i}\mu_{j}^{*}}{\mu_{i}+\mu_{j}^{*}}\frac{v_{i}-v_{j}^{*}}{r_{ij}}\frac{\partial W_{ij}}{\partial r_{ij}}\;\mathrm{if}\;x_{j}\in \Omega_{G}^{P}\;\mathrm{and}\;\forall x_{i}\in \Omega_{f}\\ \left[\frac{1}{n_{i}^{2}}+\frac{1}{n_{j}^{2}}\right]\mu_{i}\frac{v_{i}-v_{j}^{*}}{r_{ij}}\frac{\partial W_{ij}}{\partial r_{ij}}\;\;\;\;\mathrm{if}\;x_{j}\in \Omega_{G}^{D}\;\mathrm{and}\;\forall x_{i}\in \Omega_{f} \end{array}\right.$$

and

(15) $$F_{ij}^{P}=\left\{\begin{array}{c} \left[\frac{p_{i}}{n_{i}^{2}}+\frac{p_{j}}{n_{j}^{2}}\right]\frac{\partial W_{ij}}{\partial r_{ij}}\frac{x_{i}-x_{j}}{r_{ij}}\;\mathrm{if}\;x_{j}\in \Omega_{f}\;\mathrm{and}\;\forall x_{i}\in \Omega_{f},\\ \left[\frac{p_{i}}{n_{i}^{2}}+\frac{p_{j}^{*}}{n_{j}^{2}}\right]\frac{\partial W_{ij}}{\partial r_{ij}}\frac{x_{i}-x_{j}}{r_{ij}}\;\mathrm{if}\;x_{j}\in \Omega_{G}\;\mathrm{and}\;\forall x_{i}\in \Omega_{f}. \end{array}\right.$$

In Equations (14) and (15), the particle number density $n_{i}$, which satisfies $n_{i}=1/V_{i}$, is used. The effective viscosity is represented by the harmonic mean of two neighboring particles $P_{i}$ and $P_{j}$, while the pressure interaction forces, which approximate the pressure gradient, are calculated by an antisymmetric gradient stencil to satisfy the conservation criterion $F_{ij}^{P}=-\;F_{ij}^{P}$. The equations are evaluated for different cases in which the neighbor particle $P_{j}$ either is a fluid particle ($x_{j}\in \Omega_{f}$, case A) or ghost particle ($x_{j}\in \Omega_{G}$, case B) and therefore part of a periodic boundary or part of a (solid) Dirichlet boundary. For the latter case ($x_{j}\in \Omega_{G}$), a replicated viscosity $\mu_{j}^{*}$, a fictitious particle velocity $v_{j}^{*}$ (both Equation (14)) and a fictitious particle pressure $p_{j}^{*}$ (Equation (15)) is used to ensure no-slip and no-penetration boundary conditions. Further details are again out of the scope of the presented publication and can be found in [38,42,43].

### 3.2. SPH for Suspension Flow (Case C)

**Figure 2:**
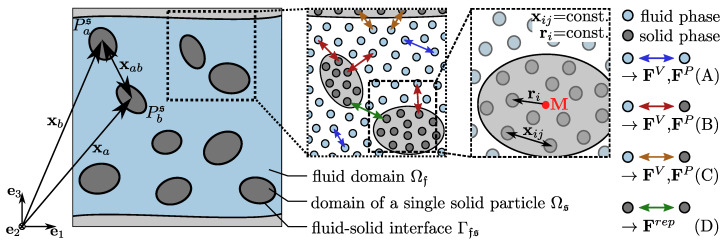
Discretization of the fluid (blue) and solid (dark grey) phase using Smoothed Particle Hydrodynamic (SPH) particles and occurring short-range particle interaction forces related to the four introduced cases (A–D).

After introducing the discretization for single-phase fluid flow, the solid–fluid interactions (case C) will be derived for the usage of SPH. Every solid particle $P^{s}$ can be discretized by a rigid collection of solid SPH particles with a no-slip and no-penetration boundary condition at all fluid–solid interfaces $\Gamma_{\mathrm{fs}}$ (Figure 2).

**Figure 3:**
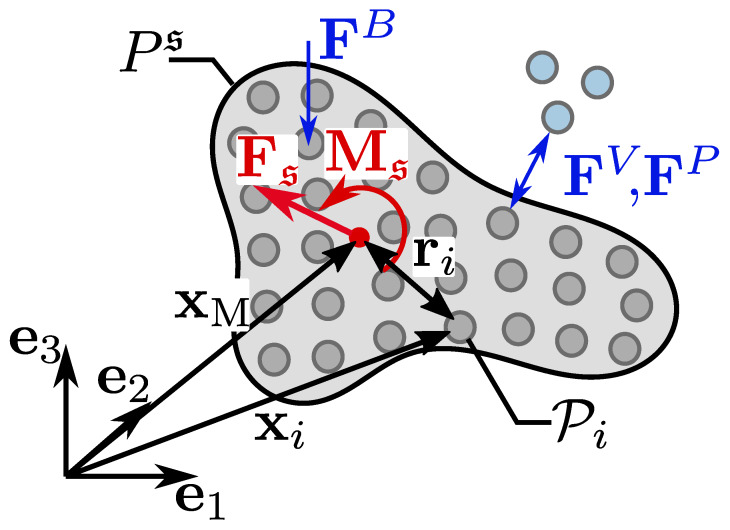
Forces acting on solid body $P^{s}$ and resulting force $F_{s}$ and moment $M_{s}$.

Doing so, earlier introduced particle forces ($F_{i}^{B}$) and short-range interaction forces ($F_{i}^{V}$, $F_{i}^{P}$) can be calculated for all solid SPH particles with fluid neighbors (see also blue arrows in Figure 3). Since solid bodies are rigid, containing solid SPH particles $P_{i}$ can not move independently. Therefore one can examine a total force $F_{s}$ (based on the balance of momentum in Equation (13)) and a total torque $M_{s}$ acting on every solid particle $P^{s}$ arose by the surrounding fluid particles.

(16) $$\begin{array}{cc} F_{s} & =F_{i}^{\mathrm{SPH}}=\sum_{i}^{n_{s}}F_{i}^{V}+\sum_{i}^{n_{s}}F_{i}^{P}+F_{i}^{B} \end{array}$$

and

(17) $$\begin{array}{cc} \;M_{s} & =\sum_{i}^{n_{s}}r_{i}\times F_{i}^{SPH}. \end{array}$$

Viscous and pressure interaction forces, $F_{i}^{V}$ and $F_{i}^{P}$, respectively, are summed up for the amount of containing SPH particles $n_{s}$ inside a solid body and $r_{i}=x_{i}-x_{M}$ is the distance of each solid SPH particle to the body’s center of mass (see Figure 3). Using the moment of inertia $J_{M}$ of a solid body

(18) $$\begin{array}{cc} J_{M} & =\int_{M}\left[\left(\bar{x}\cdot \bar{x}\right)I-\left(\bar{x}⊗\bar{x}\right)\right]\;dm \end{array}$$

(19) $$\begin{array}{cc} \; & =J_{ij}\;e_{i}⊗e_{j}=\left[\left({\bar{x}}_{1}^{2}+{\bar{x}}_{2}^{2}+{\bar{x}}_{3}^{2}\right)\;\delta_{ij}-{\bar{x}}_{i}\;{\bar{x}}_{j}\right]\;m_{i}\;e_{i}⊗e_{j}\; \end{array}$$

where the translational and angular acceleration (${\dot{v}}_{a}$, ${\dot{\omega }}_{a}$) and velocity (${\tilde{v}}_{a}$, ${\tilde{\omega }}_{a}$) of a solid particle $P_{a}^{s}$ can be calculated by

(20) $$\begin{array}{ccc} \; & {\dot{v}}_{a}=F_{s}^{a}/m_{a}\;,\; & {\tilde{v}}_{a}={\dot{v}}_{a}\;\Delta \;t\;, \end{array}$$

(21) $$\begin{array}{ccc} \; & {\dot{\omega }}_{a}={\left(J_{M}^{a}\right)}^{-1}\cdot M_{s}^{a}\;, & {\tilde{\omega }}_{a}={\dot{\omega }}_{a}\;\Delta \;t\;, \end{array}$$

and finally the total velocity $v_{i}$ of each SPH particle $P_{i}$ of the solid body can be updated to

(22) $$\begin{array}{c} v_{i}={\tilde{v}}_{a}+{\tilde{\omega }}_{a}\times r_{i}\;. \end{array}$$

The implementation generally provides no-slip and no-penetration boundary conditions at all fluid–solid interfaces $\Gamma_{fs}$. Thus fluid velocity components ($v_{f}=0$) as well as the relative velocity between solid and fluid phase states to be zero ($v_{f}=v_{s}$) on $\Gamma_{fs}$.

### 3.3. Solid–Solid Interactions in SPH (Case D)

While simulating solid particles immersed in a fluid, also solid bodies $P_{a}^{s}$ and $P_{b}^{s}$ will interact with each other. To regard this, an additional term was included into the momentum balance Equation (9). The repulsive force is discretized as presented in Equation (8) taking into account $x_{ab}=\left(x_{b}-x_{a}\right)/x_{ab}$. Based on that, the discrete balance of momentum (Equation (13)) is updated to

(23) $$m_{i}{\dot{v}}_{i}=F_{i}^{SPH}=\sum_{j}F_{ij}^{V}-\sum_{j}F_{ij}^{P}+F_{i}^{B}+F_{ab}^{\mathrm{rep}}\;.$$

Moreover the computation of the total force $F_{s}$ is extended by the addition term of the repulsive force

(24) $$\begin{array}{c} F_{s}=F_{i}^{\mathrm{SPH}}=\sum_{i}^{n_{s}}F_{i}^{V}+\sum_{i}^{n_{s}}F_{i}^{P}+F_{i}^{B}+\sum_{a\neq b}F_{ab}^{\mathrm{rep}}\;, \end{array}$$

while the calculation of the total torque $M_{s}$ remains the same as in Equation (17).

## 4. Validation of Flow with Suspended Particles

### 4.1. Details of the Implementation

As already mentioned, the presented SPH approach is implemented using the HOOMD-blue [21,22] library where packages like particle initiation and neighbor search algorithms were efficiently implemented.

**Figure 4:**
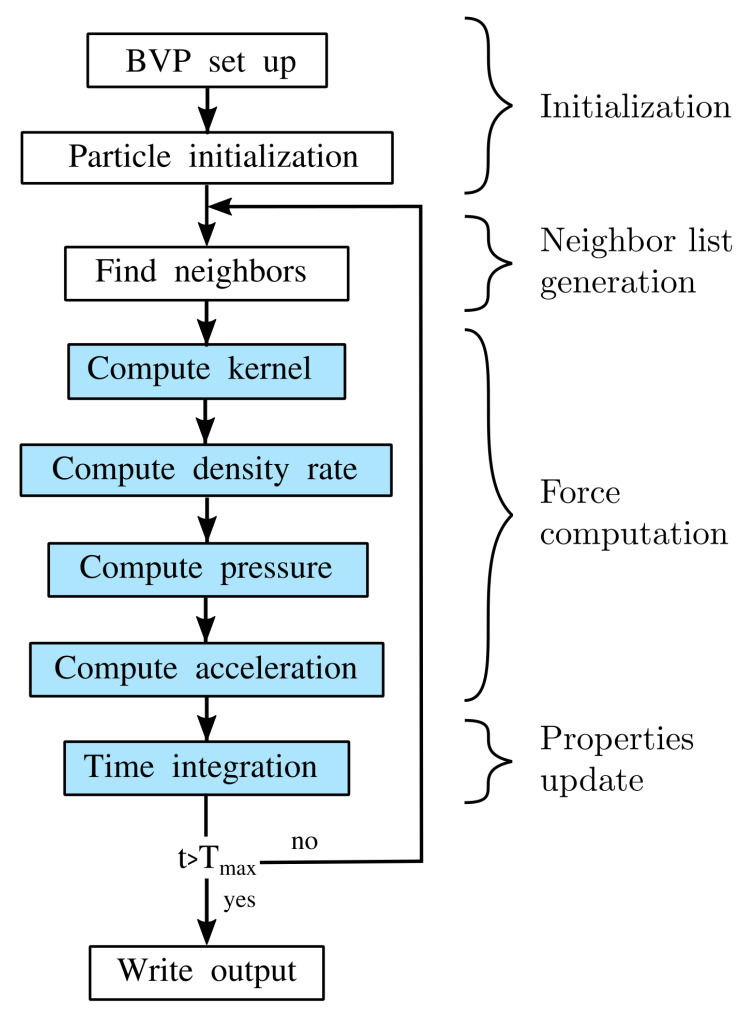
Workflow of SPH implementation. Blue background indicates own implementation within the HOOMD-blue library [23].

The implementation was validated and underwent a scalability study for a variety of representative test cases for single phase flow in porous media running on massive parallel CPU- and GPU-clusters [23]. Figure 4 shows the workflow of the SPH implementation in HOOMD-blue. After initiating the system, the implementation starts with generating neighbor lists using a cell list algorithm. Afterwards, the implemented SPH scheme computes values for the kernel, density rates, pressure and finally acceleration for each SPH particle. All here presented simulations use a fourth order Wendland kernel representation with compact support κh=3.4dx [44], where dx is the initial particle distance. The model introduced in Section 3 and underlying equations (Equations (13) and (23)) are evaluated in the force computation step, i.e., by computing the acceleration of each SPH particle. To update particle properties, a Velocity Verlet algorithm [45] is used for time integration, since it has been employed in particle methods before and exhibits good stability properties. The time step size Δt is limited by stability conditions shown in Equation (25) (following Morris [20]) to ensure stable time integration in the presence of pressure waves, viscous diffusion fronts or gravity waves. The index *i* for the max and min operators apply to all containing fluid SPH particles.

(25) $$\begin{array}{c} \Delta t\leq \min\left\{\frac{0.25h}{\max_{i}c_{i}},\frac{h^{2}\min_{i}\rho_{0,i}}{8\max_{i}\mu_{i}},\sqrt{\frac{h}{16\;\|F_{i}^{B}\|}}\right\} \end{array}$$

### 4.2. Validation of Immersed Particle Flow

While simulating suspended solid particles, objects move translational and rotational. As presented in the work of Jeffery [9], non-spherical objects with a rotational symmetry, which are immersed in a fluid under shear flow, perform rotational motion around the axis perpendicular to the shear direction. This movement is periodic and therefore Jeffery derived the tumbling rate $\dot{\varphi }$ as well as the period of rotation *T*. Following Mueller et al. [29], who gives a good overview of the derived equations and the rheology of suspended solid particles, one can reduce any rotational symmetric body to an equivalent spheroid with an aspect ratio of the rotaional half-axis $l_{a}$ to the perpendicular half-axis $l_{b}$ that can be calculated by $r_{e}=l_{a}/l_{b}$ (originally showed by Brenner [46]). Using this, Jeffery’s orbit can be computed by

(26) $$\begin{array}{c} \dot{\varphi }=\dot{\gamma }\frac{1}{r_{e}^{2}+1}\left(r_{e}^{2}\;\cos^{2}\varphi +\sin^{2}\varphi \right), \end{array}$$

and the related period of rotation by

(27) $$\begin{array}{c} T=\frac{2\pi }{\dot{\gamma }}\left(r_{e}+\frac{1}{r_{e}}\right), \end{array}$$

where φ∈{0,2π} is the vorticity (orientation) of the particle and $\dot{\gamma }$ is the shear rate.

**Figure 5:**
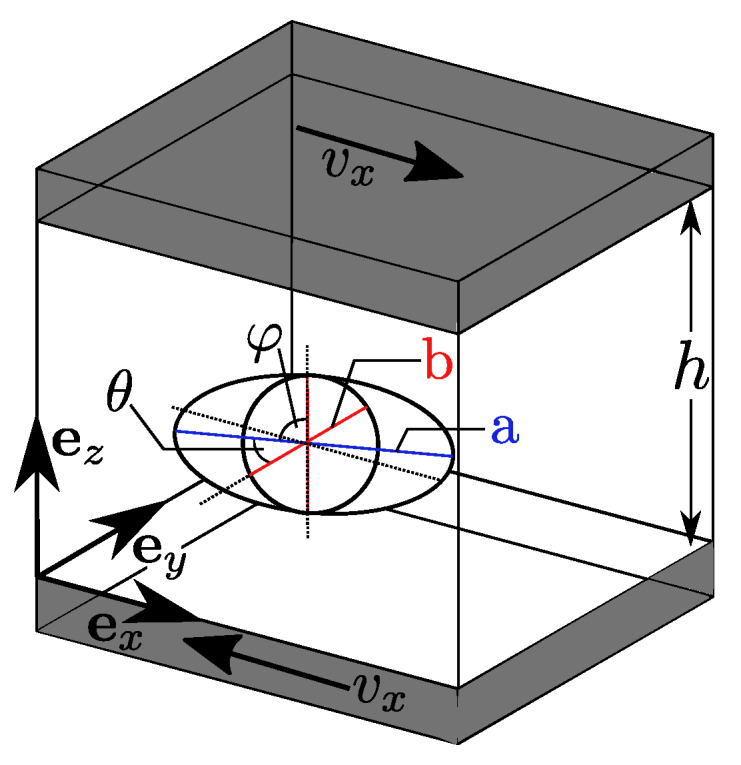
Sketch of validation test case with definition of half-axis and rotation angles as presented by Mueller [29].

This benchmark should be the basis of the validation of the presented SPH approach for suspended objects. In contrast to Jeffery’s original assumption, we choose a small but finite Reynolds number in our numerical simulation, cf. Table 1. Simulations for moderate Reynolds numbers are still rare but show the predictive power of Direct Numerical Simulations even for single non-spherical particles immersed in a fluid. We simulate three different spheroids under shear flow. The initial particle orientation is described by angles θ and φ. θ is the angle between the a-axis and the $e_{y}$-axis and φ is the angle between the plane containing the a-axis and the $e_{y}$-axis and the plane that contains the $e_{y}$- and $e_{z}$-axis as it is shown in Figure 5.

**Figure 6:**
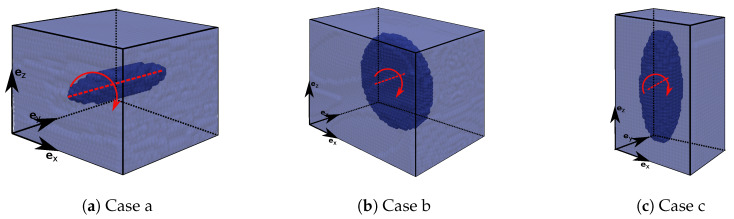
Initial configurations and rotation axis and direction of the three considered cases (**a**–**c**).

Details of the simulation input as the aspect ratio, initial orientation ($\theta_{0}$, $\varphi_{0}$) and Reynolds number (Re) are listed in Table 1. The three investigated cases (also shown in Figure 6) present both, oblate ($r_{e}<1$) and prolate ($r_{e}>1$) spheroids with initial states of the a-axis in $e_{y}$-direction ($\theta_{0}$ = 0) and $e_{z}$-direction ($\theta_{0}$ = π/2). Simulations are performed with an initial shear rate of $\dot{\gamma }=0.2\;s^{-1}$ due to motion of upper and lower wall particles with a constant velocity $v_{x,0}=0.001\;m/s$. We choose a discretization of 50 fluid SPH particles over the height h=0.01m. This leads to a discretization between 10 and 15 particles over the a-axis of the spheroid and approximately 190,000 SPH particles. Initial density was chosen to $\rho_{0}^{f}=1000\;\mathrm{kg}/m^{3}$ and initial viscosity is computed as a function of the Reynolds number by μf=ρ0fvx,0h/Re.

**Figure 7:**
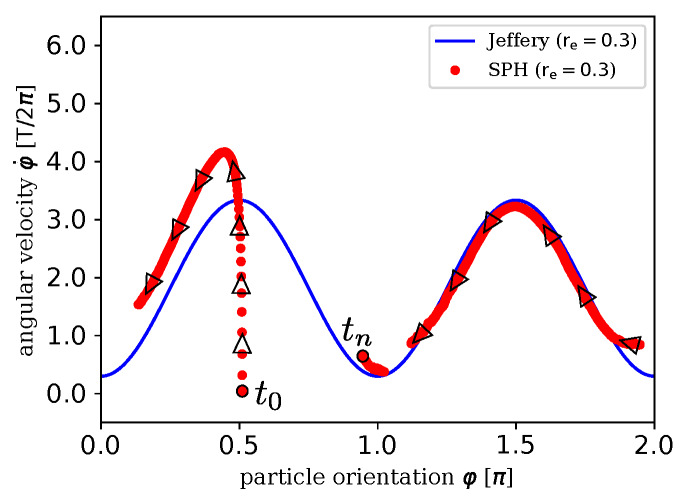
Normalized angular velocity $\dot{\varphi }$ over rotation angle φ. Comparison of SPH results (simulation **c**) with analytical solution by Jeffery [9] for $r_{e}=0.3$. Black arrows additionally show the parameter of time from the start of the simulation at $t_{0}$ to the end $t_{n}$.

Simulation results are analyzed in terms of motion, especially rotation, around the $e_{y}$-axis. Jeffery’s approach [9] (resumed by Mueller [29]) from Equations (26) and (27) considers spheroids as in case **c**. Thus, the rotation of the spheroid over its angular velocity is plotted in Figure 7. As displayed there, the measurement of the rotation in our simulation starts at a point with an angle of φ=π/2 and then rotates towards φ=0 which equals φ=2π. After that, the particle continues to rotate to an angle of φ=π. Even in one single period, the simulation results are in good agreement with the theoretical solution. Differences, especially in the first quarter of rotation (0<φ<π), are expected with regard to transient, i.e., instationary phenomena, and due to the fact that the solid particle does not only rotate but also moves translatoric due to solid–fluid interaction at the beginning of the process. Moreover, considering the factor of time (indicated by black arrows), the simulation starts at φ=π/2 and the particle is accelerated first until it reaches the theoretically rotation velocity.

**Figure 8:**
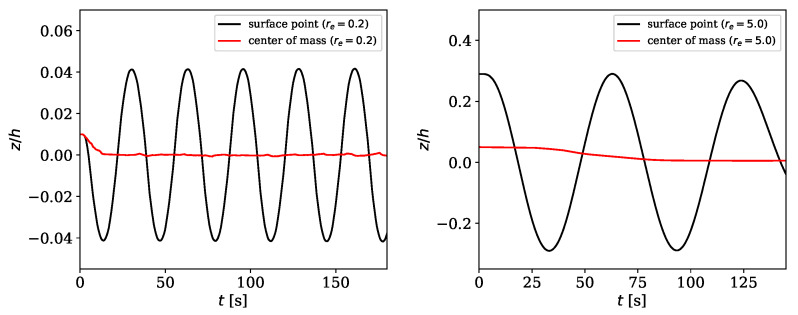
Motion of $e_{z}$-coordinate over time *t* for two different aspect ratios (case **a**: $r_{e}=0.2$, case **b**: $r_{e}=5.0$).

For cases **a** and **b** we observe a rotational motion as well (see Figure 8). Since Jeffery’s approach does not map particle rotation, when the rotation axis of the object is perpendicular to the shear direction (θ=0), a different representation of the rotation was used. Thus, the evolution of the coordinate in $e_{z}$-direction (*z*) of a surface point normalized by the channel height *h* was plotted over the time *t*. As solid particles were not placed right in the center of the channel, the particles move translatoric as well. Figure 8 shows not only the (harmonic) motion of the material point at the surface of the particle, but also the (subtracted) translatoric motion of the center of mass. It could be clearly observed, that there is a stable periodic motion for spheroids with different aspect ratios where the initial state sets the a-axis parallel to the $e_{y}$-axis (θ=0). In addition to (stationary) Jeffery orbits, the numerical simulations predict also transient effects if moderate Reynolds number have been chosen and the reference configuration of the center of mass of the immersed particle is not in the vertical symmetry plane.

**Figure 9:**
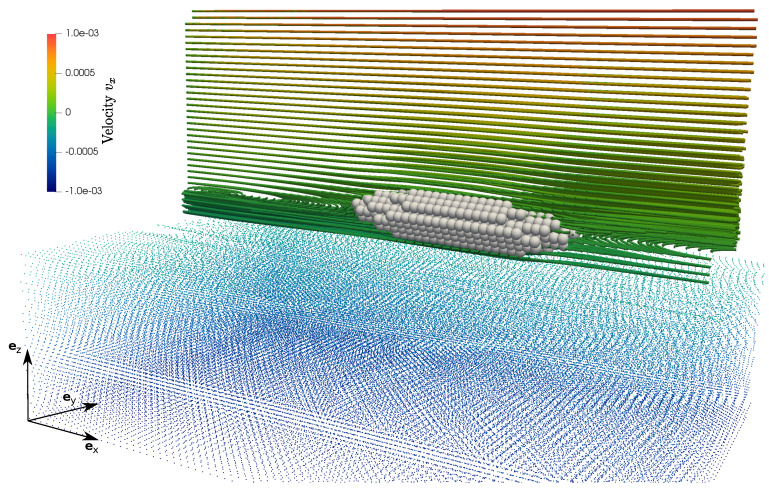
3-dimensional view of the simulation results showing streamlines of the velocity $v_{x}$ in the channel. Prolate spheroid is aligned with $e_{x}$-axis and does not perform any rotation for a Reynolds number of Re = 10.

Additionally, simulations with initial states where the a-axis of the spheroid is aligned to the $e_{x}$-axis were performed using as well a Reynolds number Re = 10. Again, we therefore expect an influence regarding to inertia effects. Indeed, the solid particle stays in the centerline region and does not perform any rotation (see Figure 9). This confirms the effect of shear induced particle migration where particles tend to move to regions with lower shear rates [6,7].

Aim of this section was to show first numerical results which are comparable to low-Reynolds number solutions validating the proposed numerical model. Due to the finite size of the computational domain, the chosen finite Reynolds number and the restricted computational time, we could not expect to get limit values of Jeffery. Nevertheless, we show a good agreement with Jeffery’s solution for the simulation with the lowest Reynolds number (Re = 1, case **c**). A detailed investigation of the Reynolds number-dependent motion of particles would be computationally challenging for Re < 1 regarding to the explicit nature of the presented SPH code.

## 5. Numerical Analysis of Solid Particles Immersed in a Fluid

**Figure 10:**
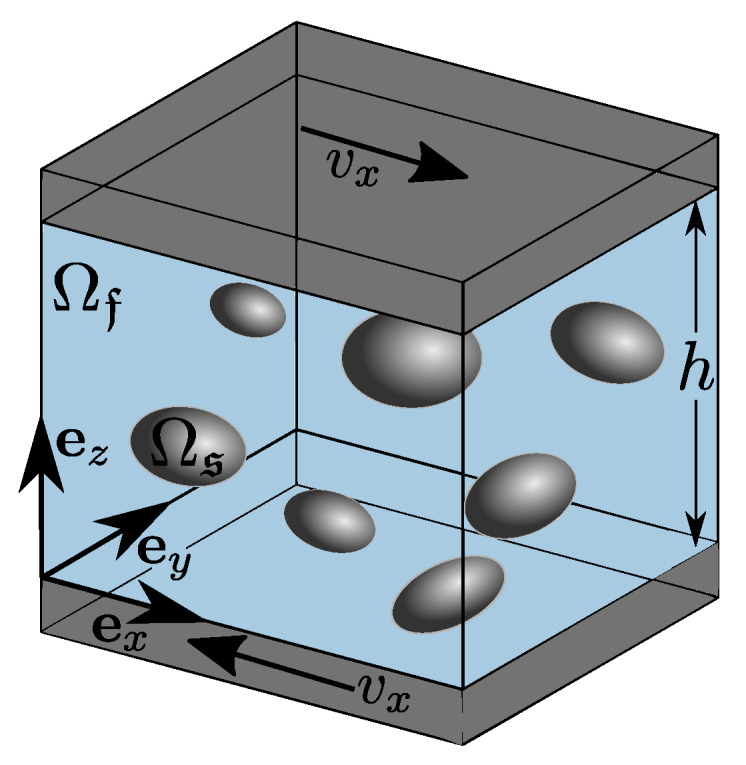
Sketch of simulated boundary value problem (BVP) of shear induced suspension flow in a channel of height *h* in $e_{z}$-direction. The simulation domain contains periodic boundary conditions in direction of $e_{x}$ and $e_{y}$. Upper and lower wall particles are moved with a constant velocity $v_{x}=0.005\;m/s$ corresponding to an input shear rate of $\dot{\gamma }=2\left|v_{w}\right|/h=0.5\;\;s^{-1}$.

The presented model is used to perform 3-dimensional, fully resolved Direct Numerical Simulations of a boundary value problem (BVP) of fluid flow containing various solid particles in a channel as shown in Figure 10. The scope of this contribution is the presentation and discussion of the chosen constitutive equation for particle-particle interactions and the related motion of a particle immersed in a fluid as well as its implemementation in SPH.

In order to investigate the effect of particle-particle interactions, we discuss the near-field solution of solid particles, e.g., of the velocity field of the fluid phase, and discuss the influence of the shape of particles as well as of the presence of other particles (with and without contact). The investigated BVPs present examples for a first numerical analysis of hydromechanical forces and contact forces. i.e., how does fluid flow and solid–solid contact influence the particle motion and vice versa.

The simulation domain consists of the fluid sub-domain which is periodic in $e_{x}$- and $e_{y}$-direction. It is limited by solid particle layers (walls) in direction of $e_{z}$. The channel has a height h=0.02m which is chosen to be the characteristic reference length $L_{\mathrm{ref}}$. We select a fourth order Wendland kernel representation with a resolution of 60 particles over the channel height. Thus, this results in an initial particle distance $dx=L_{\mathrm{ref}}/60$. In our numerical investigations, wall particles are moving with a constant velocity $v_{x}=0.005\;m/s$ such that the Reynolds numbers stay constant as Re=100. Initial viscosity of the fluid phase is chosen to be $\mu^{f}=0.001\;\mathrm{Pa}/\;s$. The initial density of the fluid is $\rho_{0}^{f}=1000\;\mathrm{kg}/m^{3}$.

Since we are interested in investigating the flow behavior dependent on a small number of immersed and interacting particles, we perform simulations with various numbers of solid aggregates (two or four solid bodies) as well as with various aggregate forms (spherical and ellipsoidal). As presented in Table 2, diameters vary between $0.05\;L_{\mathrm{ref}}$ and $0.20\;L_{\mathrm{ref}}$ (corresponding to a number of 200–800 solid SPH particles per solid particle).

## 6. Discussion of Relevant Model Parameters

The simulation results are analyzed in terms of resulting (steady state) velocities and motion of the solid bodies. Additional to a whole domain analysis, we consider fluid flow around solid bodies more closely and discuss influence of hydromechanical forces and contact forces.

**Figure 11:**
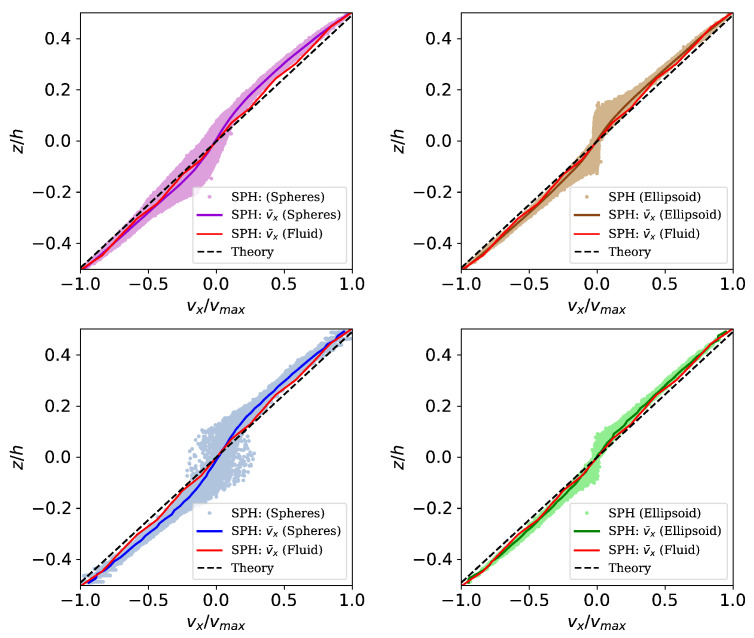
Resulting profile of velocity in $e_{x}$-direction over coordinate $e_{z}$ for spherical solids (**left**) and ellipsoidal solids (**right**). While the black line represents the analytical solution for simple shear flow of a Newtonian fluid, the red line represents the mean velocity of a simulation of shear flow without solid aggregates (only fluid). Colored markers and and the same colored line represent SPH particle velocities and mean particle velocity, respectively. Investigated cases are shear flow with two spherical (pink, **top left**), two ellipsoidal (brown, **top right**), four spherical (blue, **bottom left**) and four ellipsoidal (green, **bottom right**) solid bodies.

Figure 11 presents the resulting velocity in $e_{x}$-direction over coordinate $e_{z}$ for the shear induced flow of a suspension. The simulation results in general show a good agreement with the analytical solution for the steady state of simple shear flow of a single phase fluid (dashed black line). Since the analytical solution represents the flow of a fully Newtonian fluid, the best approximation is given in the simulation without solid bodies (red line). Simulations containing solid bodies exhibit a plateau of zero velocity in the centerline region of the simulation domain due to the there accumulated solid aggregates. Simulation results show that the numerical model is capable of reproducing the effect of shear wall migration, where solid aggregates move to regions with lower shear rates [6,7].

For an better overview, results of spheres and ellipsoids are considered in seperate plots (Figure 11 left and right respectively). Nevertheless, comparing both sides, it can be observed that the region of zero velocity remains to be wider for spherical aggregates compared to ellipsoidal aggregates. From our point of view, one reason for that is the form of the solid aggregates. While the spherical particles rotate and move due to shear induced flow, even when they already reached the centerline zone, ellispoidal particles move to the centerline wihle rotating until their longest half axis is parallel to the $e_{x}$-axis and then stay in this mode (as discussed in Section 4).

**Figure 12:**
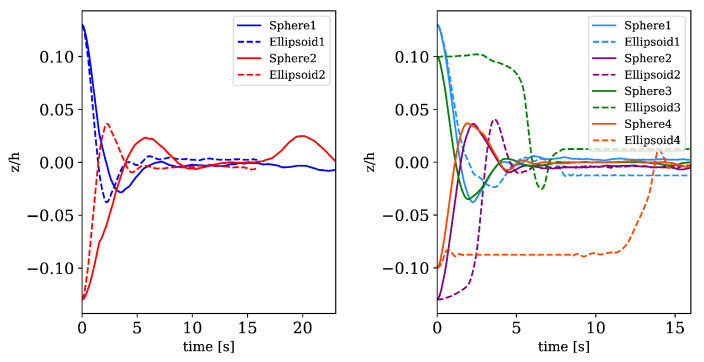
Motion of the center of masses over time for simulations containing two and four solid particles. Solid and dashed lines represent spherical particles and non-spherical particles, respectively.

Additionally, to the flow velocity, the motion of the center of mass (COM) of solid bodies is considered and analyzed. In Figure 12 we compare the motion of the COM for simulations with two solid aggregates and with four solid aggregates (spherical vs. ellipsoidal) where the initial position remains the same and only the form changes. Simulations are performed until suspensions are almost in a stationary regime (approx. 20 s), while simulations without solid particles reach a steady state much faster. We observe an oscillation around the centerline region (z=0) in the motion of all solids until they arrange themselves in this line. The motion is influenced by inertia as well as contact forces. Due to that, spherical particles (straight line) move to the centerline region and and then move with the fluid without oscillating, since their profile presents more resistance than the ellipsoidal profile. Thus, ellipsoidal particles move while rotating to the position with smallest drag. Nevertheless the results show that even with only a small number of solid bodies effects like shear-induced particle migration (as observed in experiments [47]) become visible. The fully resolved SPH simulations of this simple application examples are used to reproduce and investigate phenomena like de-mixing and migration processes. Nevertheless we do not aim to perform a full analysis of the flow regimes as for example done by Vázquez-Quesada et al. [24,25].

Even in systems with a low number of solid bodies, particle collisions occur. However, the influence of solid–solid contact is attenuated by hydromechanical forces in lubrication layers between the solid particles. Since in SPH a small layer of fluid SPH particles is mostly available between solid particles, a direct particle-particle contact does not occur. This was the main reason for choosing a repulsive force model, where the contact force scales with the distance. Therefore, an influence of hydromechanical forces during the simulation containing four ellipsoidal particles and the influence of contact forces during the simulation containing four spherical solids is analyzed.

**Figure 13:**
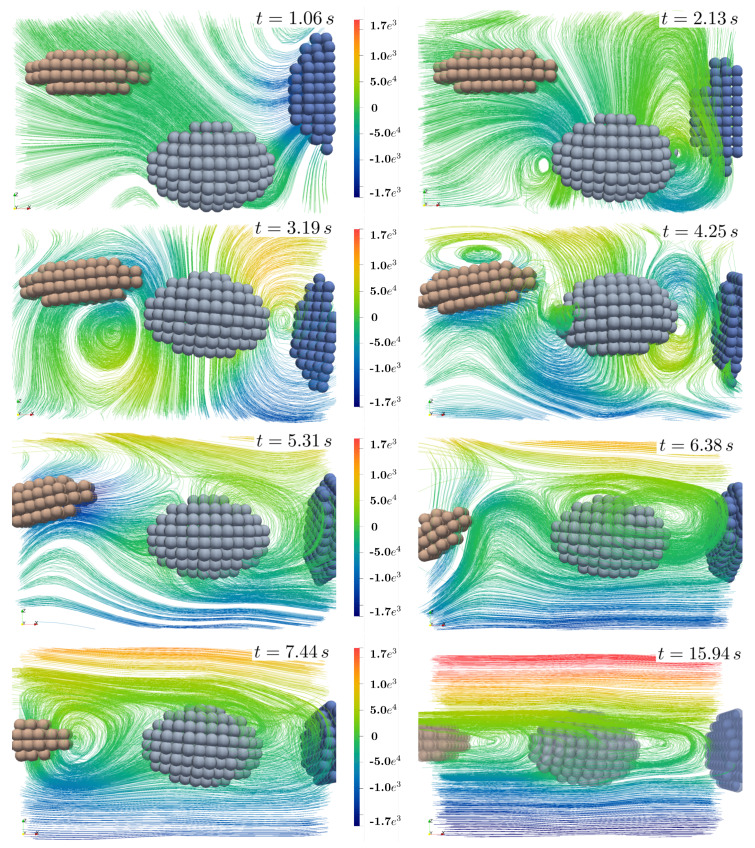
Streamlines of the velocity $v_{x}$-direction at various time steps considering the $e_{x}$-$e_{x}$-plane for simulation containing four ellipsoidal solid particles.

**Figure 14:**
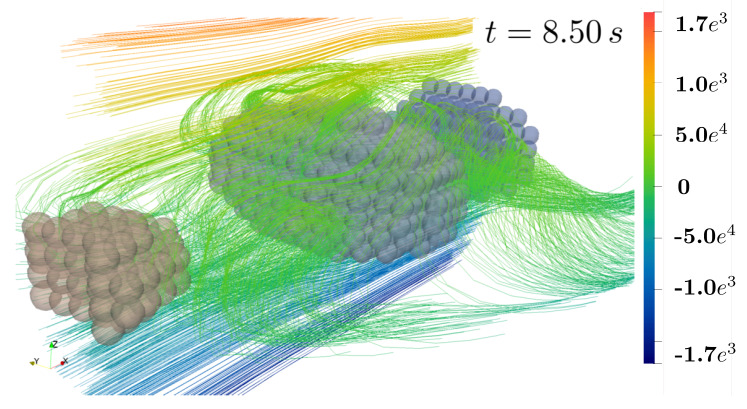
3-dimensional view of streamlines at timestep t=8.5s.

Figure 13 and Figure 14 show streamlines of the velocity in $e_{x}$-direction ($v_{x}$) in the $e_{y}$-$e_{z}$-plane and, for a better visualization, as a perspective view, respectively. Induced by surface coupled hydromechanical forces, fluid motion causes motion and rotation of solid particles until they are aligned in the centerline region and their main (largest) axis is aligned to the $e_{x}$-axis. Using moderate Reynolds numbers (Re = 100), also chaotic motion including vortex formation takes place. In this example, no direct contact between solid aggregates occur. However, motion of the non-spherical particles influence the fluid flow as well, since they stay in the center with zero velocity and therefore slow down fluid flow different to flow without or with spherical bodies (compare as well Figure 11).

**Figure 15:**
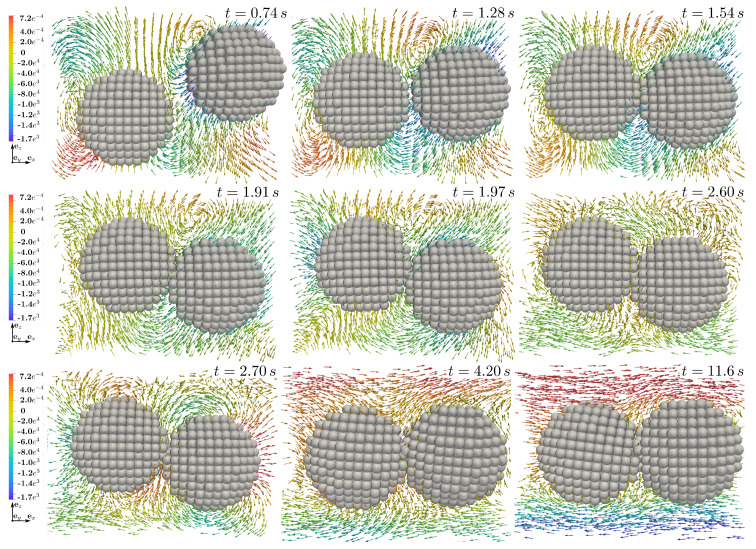
Velocity trajectories of $v_{x}$ for the evolution of contact between two solid particles over time (top left to bottom right). Contact occurs at t=1.91s and again at t=2.60s. Due to hydromechanical and hydrodynamical forces, particles stay close to each other even after contact.

Additional to hydromechanical forces, simulations with four spherical aggregates show the impact of contact between two solid particles. In Figure 15 the beginning of motion, induced by hydromechanical forces is shown. A subsequent motion towards each other, as arrows at time step t=1.28s and t=1.54s can be observed. Following this, the first contact takes place at time t=1.91s. Comparing this and the next time step (t=1.97s), one can observe the change in the flow field in the vicinity of the particles. This occurs for example at the left side of the left particle and at the lower right side of the right particle, where velocity trajectories turn in opposite directions after the contact. Nevertheless, the contact force is not high enough to separate solid bodies as they stay close to each other during the following time steps until there is a second contact recognized at t=2.60s. Again velocity trajectories react due to the solid body motion and change their direction (t=2.70s). Finally hydrodynamical forces enforce fluid flow around both bodies as a unit and not forming recirculation zones between and separating them as observed in Figure 13. From our point of view this is a result of the difference in the form of the solid particles, since at least in this simulation with bi-disperse spherical particles, they stay closer together than ellipsoidal particles, where due to larger aspect ratios and related flow profile, vortexes form much faster.

## 7. Conclusions

The current contribution presents a mesh-less Lagrangian approach aiming for Direct Numerical Simulations of the motion of immersed solid particles (grains) in a Newtonian carrier fluid. Underlying conservation equations for linear momentum of single phase flow were derived and a contact force was included into the model. In contrast to established DEM-CFD models, the fluid–solid momentum exchange is captured via interfacial forces between the solid and the fluid SPH particles. The model was implemented in an explicit quasi-incompressible SPH scheme and validated in terms of periodic motion of single non-spherical solid particles in shear flow at moderate Reynolds numbers. Boundary value problems of spherical and ellipsoidal solid particles were investigated. Resulting velocity profiles of dominating velocity $v_{x}$ have been analyzed and compared with each other and, additionally, with solutions of single-phase fluid flow. The numerical results predict that flow with immersed solid particles differs to single-phase fluid flow mainly in the centerline region (z=0) where the flow velocity is zero. Due to hydromechanical forces the aggregated particles are accelerated and migrate to this region where they stick. A detailed analysis of the flow field in the vicinity of solid particles shows, that dependent on the aggregates form, vortexes form between the aggregates influencing further motion, while ellipsoids promote the formation and spherical particles tend to build formations so that no vortexes occur in between.

Concentrating on microhydrodynamical effects, we simulate only a small number of immersed particles. Well known effects, like the movement of solid aggregates towards regions with lower shear rates, reported by Chun [6] and Shauly [7] as well as general shear-induced particle migration, as reported by Husband [47], are reproduced. However, the number of solid particles is to small to investigate larger scale phenomena phenomena like de-mixing as observed in [3]. Therefore future investigations will include simulations with more solid particles to analyze these effects in further detail.

To come closer to technical applications, like for example concrete pumping processes, we will investigate larger-scale BVPs, where fluid-flow is induced by a volumetrical force corresponding to a pressure difference. Interesting details are the migration of particles dependent on their form and size as observed by Fataei et al. [8] as well as the evolution of flow profiles dependent on the volume fraction of solid aggregates as discussed by Ivanova et al. [48]. Ongoing research moreover includes the implementation of a lubrication correction to stabilize the model by correcting hydro-dynamic forces acting between two solid particles as proposed by Vazquez-Quesada and Bians [4,5].

## Figures and Tables

**Table 1:** Summary of parameters of spheroids for the three different simulation scenarios.

	$r_{e}$	Re	$\theta_{0}$	$\varphi_{0}$
**a**	0.2	10.0	0	undefined
**b**	5.0	10.0	0	undefined
**c**	0.3	1.0	π/2	0

**Table 2:** Summary of parameters of solid aggregates for the four different simulation scenarios. Spherical solids are defined by their radius *r* while ellipsoidal solids are defined by their half-axes *a*, *b* and *c* (dimension in $e_{x}$-, $e_{y}$- and $e_{z}$-direction, respectively).

Simulation with	Parameter of Aggregates [mm]			
2 spherical solids	*r* = 3.0	*r* = 4.0		
2 elliptical solids	*a* = 3.0 *b* = 2.0 *c* = 1.0	*a* = 2.2 *b* = 4.0 *c* = 3.0		
4 spherical solids	*r* = 2.6	*r* = 2.6	*r* = 3.0	*r* = 3.0
4 elliptical solids	*a* = 2.0 *b* = 1.0 *c* = 3.0	*a* = 3.0 *b* = 4.0 *c* = 2.0	*a* = 3.0 *b* = 2.0 *c* = 1.0	*a* = 2.0 *b* = 1.0 *c* = 3.8
